# Comparison of hearing recovery criteria in sudden sensorineural hearing loss

**DOI:** 10.1590/S1808-86942012000300009

**Published:** 2015-10-14

**Authors:** Daniel Paganini Inoue, Eduardo Amaro Bogaz, Flávia Barros, Norma de Oliveira Penido

**Affiliations:** a Specialist in Otology at the Federal University of São Paulo (MSc student at the Otorhinolaryngology and Head and Neck Surgery Graduate Program of the Federal University of São Paulo).; b Specialist in Otology at the Federal University of São Paulo (MSc student at the Otorhinolaryngology and Head and Neck Surgery Graduate Program of the Federal University of São Paulo).; c MSc at the Federal University of São Paulo. (Speech and Hearing Therapist at the Sudden Deafness Ward of the Federal University of São Paulo.); d Post-doctoral degree at the Federal University of São Paulo. (Professor, Advisor and Coordinator of the Otorhinolaryngology and Head and Neck Surgery Graduate Program of the Federal University of São Paulo). Departamento de Otorrinolaringologia e Cirurgia de Cabeça e Pescoço da Universidade Federal de São Paulo.

**Keywords:** audiometry pure-tone, audiometry speech, hearing loss sudden, speech intelligibility

## Abstract

The countless methods available to analyze hearing recovery in idiopathic sudden sensorineural hearing loss (ISSHL) cases hinder the comparison of the various treatments found in the literature.

**Objective:**

This paper aims to compare the different criteria for hearing recovery in ISSHL found in the literature.

**Materials and Methods:**

This is an observational clinical cohort study from a prospective protocol in patients with ISSHL, treated between 2000 and 2010. Five criteria were considered for significant hearing recovery and four for complete recovery by pure tone audiometry, using non-parametric tests and multiple comparisons at a significance level of 5%. After determining the stricter criteria for hearing recovery, vocal audiometry parameters were added.

**Results:**

There was a significant difference between the criteria (*p* < 0.001) as they were analyzed together. Mild auditory recovery occurred in only 35 (27.6%) patients. When speech audiometry was added, only 34 patients (26.8%) showed significant improvement.

**Conclusions:**

There is a lack of consistency among the criteria used for hearing recovery. The criterion of change of functional category by one degree into at least mild hearing recovery was the stricter. Speech audiometry did not prove essential to define significant hearing recovery.

## INTRODUCTION

Sudden sensorineural hearing loss (SSHL) was originally described by DeKleyn^1^. According to the National Institute of Deafness and Other Communication Disorders, SSHL is defined as sensorineural hearing loss of at least 30 decibels (dB) occurring in at least three consecutive frequencies within 72 hours^2^.

Incidence rates range between 5 and 20 cases for every 100,000 individuals per year^3^. Therefore, it is estimated that in the city of São Paulo some 500 to 2,000 new cases occur every year. However, it is believed that a significant share of this population fails to seek medical care, once studies have shown that 32-65% of the patients improve spontaneously^4^, ^5^.

Hearing progress analysis in SSHL patients is more challenging to do than in patients with other inner ear diseases, as various other factors may be present such as vertigo and/or tinnitus, multiple compromised audiometric frequencies, previously established hearing loss or comorbidity, and varying degrees of hearing loss. Additionally, there is a limited number of patients suffering from SSHL, a disease that scarcely ever stems from one single pathologic process.

Aside from the challenges listed above, there is no universally accepted standard for hearing recovery in the studies carried out on SSHL. Plontke *et al.*^6^ looked into 52 controlled trials on the treatment of idiopathic sudden sensorineural hearing loss (ISSHL) only to find 40 different analytical methods. Numerous ways to calculate hearing recovery were also identified, using pure tone average (PTA) accompanied or not by speech audiometry parameters such as word recognition scores (PB max), auditory discrimination, and speech recognition threshold (SRT).

One of the various parameters used to assess hearing recovery is 10-dB improvements on PTA at 0.5, 1, and 2 kHz described by Stakroos *et al.*^7^ in a prospective randomized controlled double-blind trial with ISSHL patients. However, the Ministry of Health, Labor and Welfare of Japan Study Group for Sudden Deafness recommends the use of the average at 0.25, 0.5, 1, 2, and 4 kHz and considers full recovery when the PTA or the thresholds of the healthy ear has reached thresholds under 20 dB; important recovery occurs when improvements are of at least 30 dB; minor recovery when it ranges between 29 and 10 dB; and no recovery when the improvement remains under 10dB. This set of criteria was also used by Suzuki *et al.*^8^. Other authors have adopted stricter hearing recovery criteria, as is the case of Penido *et al.*^9^, for whom full recovery occurs when the PTA at 0.5, 1, 2, and 4 kHz has improved by over 90% based on a normal audiometric level of up to 25 dB; partial recovery was set between 21% and 90%; and no recovery when improvements were under 20%.

Other forms of assessment have been proposed: Wilson *et al.*^4^ have defined full recovery when the PTA at 0.5, 1, and 2 kHz or the SRT were under 10 dB; partial recovery when an improvement of at least 50% was observed in relation to the unaffected side; and no recovery when improvement was under 50%. These parameters were adopted to study the efficacy of using steroids to treat SSHL. Dallan *et al.*^10^ considered recovery as an improvement on the degree of hearing loss from the calculation of PTA at 0.5, 1, 2, and 3 kHz. Hearing loss grades were divided into normal (hearing level of at least 25 dB), mild (under 25 dB and equal to or greater than 40 dB), moderate (under 40 dB and equal to or greater than 70 dB), and severe (under 70 dB). Other studies have associated speech recognition index and pure tone average analyses, as seen in Slattery *et al.*^11^. The recovery criterion used by these authors was improvement by 10 dB or 50% on the PTA at 0.5, 1, 2, and 3 kHz or improvement of 12% on PB max.

The lack of consistency between the analytical methods used in trials on idiopathic sudden sensorineural hearing loss hinders the establishment of a proper comparison between the various treatments described in the literature. Berliner *et al.*^12^ studied stapedectomy patients to find that outcome is affected by the frequencies and principally the criteria defining the procedure as successful. Therefore, audiometric parameters should be standardized along with criteria to measure success to analyze the various treatments for ISSHL.

This study aims to compare the various criteria for hearing recovery described in the literature for patients with sudden sensorineural hearing loss seen at a university hospital.

## MATERIALS AND METHODS

This is a cohort observational trial based on a prospective protocol that included patients from the sudden hearing loss ward of a university hospital affected by idiopathic sudden sensorineural hearing loss seen between 2000 and 2010. This study was approved by the institution's Ethics Committee and given permit number 1540/08.

The protocol included patients with unilateral ISSHL of at least 30 dB in at least three consecutive frequencies occurring within up to 72 hours seen at the Sudden Hearing Loss Ward for at least two months or who recovered completely before the end of these two months. All patients included signed a free informed consent form. They were treated with oral prednisone 1 mg/kg/day (maximum daily dosage of 60 mg) for at least one week, and then had their dosages reduced on a weekly basis for up to 21 days. Patients with contraindication for the prescribed prednisone dosages had their dosages reduced or, in a few rare occasions took deflazacort instead.

Patients with history of otological disease and cases with a confirmed etiology for sudden hearing loss, i.e., patients showing conditions that may evolve together with SSHL such as trauma, infection, exposure to ototoxic drugs, barotrauma, and suspected for endemic parotitis, were excluded from the study. Patients with retrocochlear disease or malformation in the inner or middle ear detected by inner ear MRI, patients with defined Ménière's disease as per the criteria of the American Academy of Otolaryngology-Head and Neck Surgery Foundation^13^, bilateral cases, and patients starting follow-up 45 days or later after the onset of hearing loss were also excluded.

The sample was analyzed for gender, involved side, and type of audiometric curve. Curves were deemed ascending when reductions were greater than 15 dB in the worst low frequency in relation to the other frequencies; U curves were the ones in which reductions greater than 15 dB were seen in the worst medium frequencies in relation to the worst low and high frequencies; inverted-U curves occur when there are reductions greater than 15 dB in the worst high and low frequencies in relation to medium frequencies; descending curves show reductions of 15 dB in average values from 4 to 8 kHz in relation to the averages seen at 250 and 500 kHz; and flat curves occur when there are differences smaller than 15 dB between the average values seen at 250 and 500 Hz, 1 and 2 kHz, and between 4 and 8 kHz.

The auditory assessments of included patients were done by the same speech and hearing therapist. Auditory assessment was made up by pure-tone audiometry at 0.25, 0.5, 1, 2, 3, 4, 6, and 8 kHz, speech audiometry along with speech recognition threshold analysis, and impedance tests for stapedial reflexes. Initial and final audiometric parameters were analyzed, the latter being obtained at least two months after the initial audiometric assessment or earlier in cases where full recovery was observed.

Initial and final average values for pure-tone audiometry tests of every patient were collected for the sets of frequencies covered. For low and medium frequencies, average values were gathered for 0.25, 0.5, 1, and 2 kHz; for medium and high frequencies, average values were gathered for 1, 2, 3, 4, 6, and 8 kHz; for high frequencies alone, average values were gathered for 3, 4, 6, and 8 kHz; for low, medium, and high frequencies combined, average values graves of all eight frequencies were collected. Low frequencies were 250 and 500 Hz; medium frequencies were 1 and 2 kHz; high frequencies were 3, 4, 6 and 8 kHz.

When hearing thresholds for profound hearing loss were not detected, the upper limit of the device (120 dB) was considered.

Tucci *et al.*^14^ indicated the use of audiometric thresholds of the involved ear to calculate hearing recovery as a baseline, under the assumption that hearing was symmetrical before the onset of SSHL. In the calculation, the author considered only the initial PTA of the healthy ear. However, we believe that if the initial and final PTA values of the healthy ear are used we might be able to reduce both systematic and random error, once the measures for the healthy and involved ears are obtained at one same time. Thus, the following formula was used to calculate PTA-related hearing recovery in decibels:

PTA recovery (dB) = (PTAII- PTAIH)- (PTAFI-PTAFH).

PTA-related percent hearing recovery was calculated as follows:

PTA recovery (%)= (PTAII- PTAIH)- (PTAFI-PTAFH)X 100/ (PTAII- PTAIH).

Where: PTAII is the initial PTA value in the involved ear; PTAIH is the initial PTA value of the healthy ear; PTAFI is the final PTA value of the involved ear; and PTAFH is the final PTA of the healthy ear.

In order to compare the criteria to assess hearing recovery in ISSHL patients, this paper contemplates five sets of parameters described in the literature to characterize significant hearing improvement and four used to substantiate full recovery based on pure-tone audiometry. All patients categorized as fully recovered were included in the group for significant recovery cases. See below the five criteria:

• **Criterion A:** significant recovery - improvement of at least 10 dB between the initial and final PTA as described by Stokroos *et al.*^7^;
• **Criterion B:** significant improvement - improvement of at least 30 dB between the initial and final PTA or when final hearing thresholds are under 20 dB or the thresholds of the healthy ear have been reached; and full recovery - final hearing thresholds are under 20 dB or the thresholds of the healthy ear have been reached as described by Suzuki *et al.*^8^;
• **Criterion C:** significant recovery - recovery above 20% of the potential improvement; full recovery - recovery above 90% of the potential improvement as described by Penido *et al.*^9^;
• **Criterion D:** significant recovery - improvement greater than 50% on PTA or when final hearing thresholds are up to 10 dB higher than the thresholds of the healthy ear; full recovery - final hearing thresholds are up to 10 dB higher than the thresholds of the healthy ear as described by Wilson *et al.*^4^;
• **Criterion E:** significant recovery - occurrence of change in functional class and mild/normal final hearing loss; full recovery - hearing thresholds are back to normal levels. Patients with pure-tone averages below 25 dB were not considered to have hearing loss; mild hearing loss was diagnosed for PTA values ranging between 26 and 40 dB; moderate hearing loss for the 41-70 dB range; severe for the 71-90 dB range; and profound for PTA values above 90 dB as described by Dallan *et al.*^10^.

Cochran's non-parametric test was used to compare the five criteria for significant hearing recovery altogether with a significance level of 5%. In order to identify which of the criteria would stand out from others, multiple comparisons were carried out using the Student-Newman-Keuls test with a significance level of 5%. These tests helped pick the stricter hearing recovery criterion.

Statistical analysis indicated that the stricter PTA-based criterion was E (see discussion for details). Speech audiometry parameters were added to criterion E, defining that significant hearing recovery occurs when there is an improvement of at least 50% on speech recognition thresholds or when SRT was up to 10 dB above that of the healthy ear and when the initial PB max was under 50% and moved to above 50% or when the initial PB max was above 50% and improved by at least 12%. The speech recognition threshold had to be up to 10 dB lower than that of the healthy ear and the PB max above 90% or equal to that of the healthy ear to consider that full hearing recovery had occurred. These parameters for SRT were based on the paper by Wilson *et al.*^4^, while the one for speech recognition was based on Slattery *et al.*^11^. This new criterion encompassing pure tone and speech audiometry was called Criterion F.

## RESULTS

This study looked into 277 patients with sudden sensorineural hearing loss. Eight patients did not meet the standard of having hearing loss of at least 30 dB in at least three consecutive frequencies. Ten were bilateral cases and in 33 the cause of hearing loss could be established. Seventy-five patients were lost during the first two months of follow-up. Twenty-four patients declined to sign the free informed consent term. The final sample stood at 127 patients. Within the valid sample 50.4% were females and 49.6% were males. As to involved ear, 46.5% had their right ear involved and 53.5% had hearing loss in their left ears. Low, medium, and high frequencies were reached in 83.5% of the patients; low and medium in 6.3% of the cases; medium and high in 7.1%; and high alone in 3.1% of the patients. Audiometric curves were categorizes as descending in 40.2% of the cases, flat in 40.9%, ascending in 14%, U-shaped in 3.9%, and inverted-U in 0.8%. Systemic steroids were administered to 100 (78.8%) patients. The absolute average rate of recovery stood at 25.3 dB. When compared to the involved ear, the rate dropped to 23.6 dB. The average relative percent recovery was 37.2%.

As we looked at the outcomes based on each criteria analyzed, we could see that when criterion A was used, 89 (70.1%) patients were categorized as presenting hearing recovery. When adopting criterion B, significant recovery was seen on 49 (38.6%) patients and full recovery in 20 (15.7%). By the standards set in criterion C, significant improvement was seen in 82 (64.6%) patients and full recovery in 15 (18.8%). According to criterion D, 44 (34.6%) patients had partial recovery while 24 (18.8%) recovered fully. Based on criterion E, significant recovery was seen in 35 (27.6%) and full recovery in 20 (15.7%) patients.

A *p*-value < 0.001 was found in the comparison made between hearing recovery criteria A, B, C, D, and E. Therefore, the criteria were statistically different from each other (Table 1).

**Table 1:** A comparison between the criteria for hearing recovery.

	No		Yes	
	N	%	N	%
Significant Improvement Criterion A	38	29.9%	89	70.1%
Significant Improvement Criterion B	78	61.4%	49	38.6%
Significant Improvement Criterion C	45	35.4%	82	64.6%
Significant Improvement Criterion D	83	65.4%	44	34.6%
Significant Improvement Criterion E	92	72.4%	35	27.6%

Cochran's non-parametric test (*p*-value < 0.001).

*P*-values of 0.069 and 0.105 were found when comparing criteria A to C and B to D, i.e., these criteria were not significantly different from each other. However, when all criteria were compared to each other, that is, A to B, A to D, A to E, B to C, B to E, C to D, C to E, the p-value was under 0.05, thus eliciting the statistically significant difference between them (Table 2).

**Table 2:** A comparison between the criteria for hearing recovery.

	*p*-value
Criterion A x Criterion B	0,001
Criterion A x Criterion C	0,069
Criterion A x Criterion D	0,003
Criterion A x Criterion E	0,003
Criterion B x Criterion C	0,001
Criterion B x Criterion D	0,105
Criterion B x Criterion E	0,039
Criterion C x Criterion D	0,002
Criterion C x Criterion E	0,002
Criterion D x Criterion E	0,046

Student-Newman-Keuls test.

**Graph 1:**
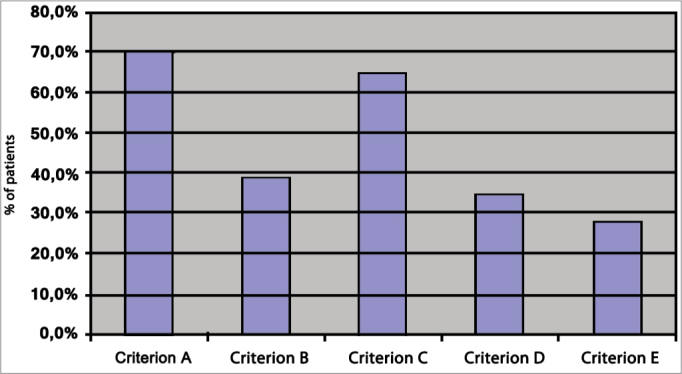
percent distribution of hearing recovery by criterion.

The percent distribution of significant hearing recovery levels defined by each of the criterion indicated that criterion E shows the lower percent improvement levels, while criteria A and C show the higher percent improvement levels (Graph 1). Therefore, criterion E was considered stricter.

**Graph 2:**
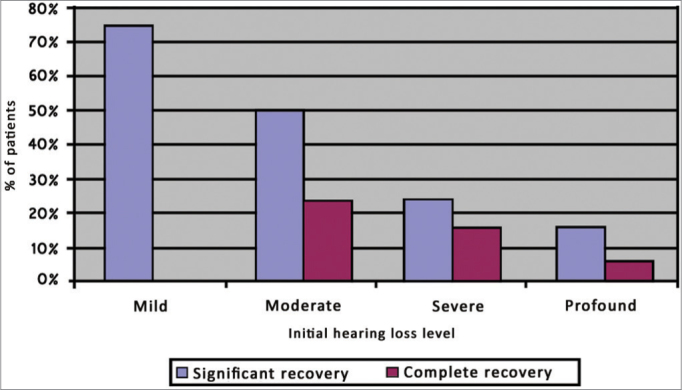
percent distribution of hearing recovery by degree of initial hearing loss based on criterion E.

Criterion E allows the stratification of recovery for each degree of initial hearing loss. Four patients were categorized as having mild hearing loss, 34 as moderate, 37 as severe, and 52 as profound. Full recovery was observed in 75% of mild hearing loss patients; among moderate cases significant recovery was seen in 50% of the patients and full recovery in 23.5%; severe patients recovered significantly in 24.3% of the cases and fully in 16.2%; profound hearing loss patients showed recovery rates of 11.5% and 5.8% for significant and full recovery respectively (Graph 2).

Criterion F revealed significant recovery in 34 (26.8%) patients and full recovery in 20 (15.7%). On average, speech audiometry parameters showed improvements of 36% in auditory discrimination and 33.2 dB in speech recognition thresholds or 37.2% of the potential recovery.

## DISCUSSION

Numerous criteria for hearing recovery were utilized in the analysis of our patients in an attempt to cover most of the parameters found in the literature, ranging from pure-tone audiometry to speech audiometry, the latter in terms of speech recognition thresholds and auditory discrimination. The criteria were adjusted to allow for comparisons between them.

No significant difference was found in the comparison made between criteria A and C and criteria B and D. Nonetheless, differences were found in all other comparison, thus showing the lack of consistency between the criteria for hearing recovery published in the literature. Byl^3^ stated that there was neither a universally accepted definition for sudden hearing loss nor en effective method to measure hearing recovery. Berliner *et al.*^12^ studied post-stapedectomy hearing recovery and stressed the relevance of adopting strong sets of criteria, as significant improvement rates depend more on the criteria than on the frequencies included in patient analysis.

Criteria A and C showed higher hearing improvement percentages than the others. However, they fail to demonstrate the reality of hearing recovery as observed in the clinical setting, once an increase of a meagre 10 dB or even of 20% on pure-tone average values will not amount to anything significant for patients with severe to profound hearing loss.

Criterion B used by Suzuki *et al.*^8^, when assessing absolute hearing recovery in decibels, fails to consider the initial degree of established hearing loss. Hearing recovery values of 30 dB in a patient with initial hearing loss of 40 dB is completely different from such recovery level in a patient with 120 dB of hearing loss. Additionally, patients with severe hearing loss present greater potential for hearing recovery. Therefore, from the clinical standpoint, this criterion is severely limited.

Wilson *et al.*^4^ calculated hearing recovery as a percentage to greatly eliminate the bias found in criterion B. However, patients with initial hearing loss of 120 dB who moved to a hearing threshold of 60 dB would qualify for the hearing recovery parameter, but would still present significant hearing loss.

Criteria B and D showed hearing recovery rates different from the ones seen in their original studies. Wilson *et al.*^4^ reported a recovery rate of 61% in patients taking steroids, while in our sample significant recovery was found in only 34.6% of these patients. Suzuki *et al.*^8^ reported a significant recovery average of 52.2% including patients on steroids or batroxobin, while our significant recovery rate remained at 38.6%. One of the possible reasons that may have contributed to this discrepancy is the fact that our study used pure-tone averages covering all affected frequencies and all higher frequencies, while the original studies used only the 0.5, 1 and 2 kHz bands and the 0.25, 0.5, 1, 2, and 4 kHz bands respectively. Lower frequencies may have a better prognosis for hearing recovery. Another factor that may have interfered with our results is that only 78.8% of our sample took systemic steroids on the prescribed dosage.

Dallan *et al.*^10^ considered that patients changing functional classes would be experiencing hearing recovery. The authors had mild, moderate, and severe hearing loss in their functional classes. Therefore, patients moving from severe to moderate hearing loss were considered to have undergone significant recovery - and 55% of them were categorized as such. In our sample that same criterion saw 51.2% of the patents as having recovered significantly - quite close to what Dallan *et al.*^10^ found. However, assuming that moderate hearing loss is not clinically acceptable, we opted for a more stringent criterion to define significant hearing recovery when the patients were able to move to a status of at least mild hearing loss.

Considering pure-tone audiometry assessment, it is our opinion that criterion E is the one closest to matching the clinical expectations around what significant hearing recovery means. The functional characterization of this criterion includes speech audiometry parameters. Yet, very little difference was observed, as only one patient who moved to mild hearing loss had an auditory discrimination score under 50%. This probably happened because criterion E has stricter hearing recovery parameters than the other methods. It might be that if the pure-tone audiometry criteria are less strict, the importance of speech audiometry as a whole will be greater. Therefore, we recommend that Criterion E be used to assess hearing recovery in ISSHL patients.

Despite our findings, we believe that all pure-tone and speech audiometry parameters should be an integral part of the assessment of sudden sensorineural hearing loss patients. Future detailed analyses of this topic will allow us to define the prognostic profile of each patient so as to offer them customized care, bearing in mind that his is an idiopathic disease with myriad etiologies. Thus, the characteristics of initial hearing loss may guide us in the adoption of specific therapeutic approaches as we are better able to tell which patients will not evolve satisfactorily from the clinical and functional standpoints.

## CONCLUSION

• There is a lack of consistency in the criteria used to verify hearing recovery in sudden sensorineural hearing loss patients; such inconsistencies hinder the comparison between research findings on the topic. Standardization is urgently required.
• The criterion that uses changes in auditory functional class to at least mild hearing loss was the one that had lower rates of significant hearing recovery of all criteria analyzed, and was thus elected the stricter of the lot.
• Speech audiometry parameters were not required to define significant hearing recovery.
